# Genome Sequences of Bacteriophages Natasha and RustyBoy, Isolated on *Arthrobacter globiformis* , and Fazel, Isolated on *Gordonia rubripertincta* in Southwestern New Mexico

**DOI:** 10.17912/micropub.biology.001988

**Published:** 2025-12-15

**Authors:** Rebecca Merchant, Violet Kirk, Isabella McClish, Meghan Ferrara, Luke Garcia, Riley Oesch, Itxel Barrera-Moncayo, Alan Blair, Erika Eaton, Gwenevere Gatto, Laurel Geoffrion, Micah Kirkman, Abey Kouchich-Martens, Lalitha Madduri, Sophia Rosa, Marisa Valdez, Ana Zamora, Linda DeVeaux, Nathaniel Jobe, Kaarin Goncz

**Affiliations:** 1 Biology, New Mexico Institute of Mining and Technology, Socorro, NM, US

## Abstract

We report the discovery and genome sequence of three phages with siphovirus morphology isolated from soil collected in Socorro, New Mexico. Phages Natasha and RustyBoy, which were isolated using *Arthrobacter globiformis* B-2979, are both assigned to actinobacteriophage cluster AW based on gene content. Phage Fazel was isolated using *Gordonia rubripertincta* NRRL B-16540 and assigned to cluster DJ.

**Figure 1. Transmission electron micrographs of phage lysate display a siphovirus morphology:**
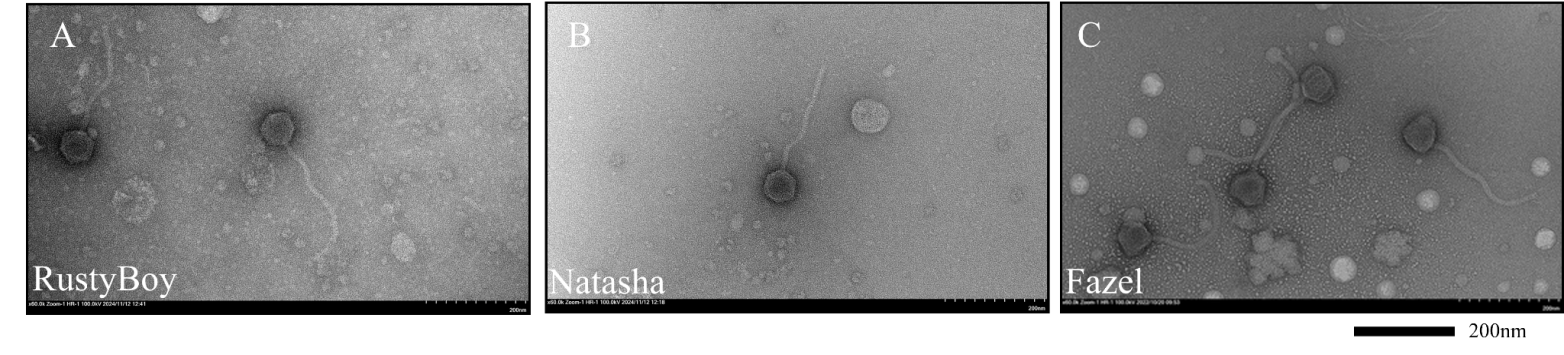
RustyBoy (A), Natasha (B), and Fazel (C). Lysates were placed on 200-mesh formvar-covered, carbon-coated copper grids (EMS) stained with 2% uranyl acetate and imaged in a Hitachi HT7800 TEM at 100 kV. Scale bar is 200 nm. Images by Tagide deCarvalho.

## Description

Bacteriophages are abundant, present across diverse environments, and are genetically diverse (Clokie et al., 2011). They have multifaceted roles in bacterial ecology and evolution (Chevallereau et al., 2022). Their isolation and genetic characterization continues to advance molecular biology, biotechnology, and medicine (Sharma et al., 2017). Here, we describe the isolation and genomic characteristics of three novel bacteriophages.

All three phages were isolated from soil samples collected in Socorro, New Mexico, (GPS coordinates provided in Table 1) using standard methods (Zorawik et al., 2024). Briefly, soil samples were resuspended in PYCa liquid medium and the suspension subsequently filtered (0.2 micron pore size). Fazel was isolated from a plaque that formed after the filtrate was plated directly in top agar with *Gordonia rubripertincta* NRRL B-16540, following incubation at 30˚ C for 3 days. RustyBoy and Natasha were isolated from plaques after the filtrate was first inoculated with *Arthrobacter globiformis* B-2979 and allowed to incubate with shaking for 72 hours at 30˚C before the resulting culture was filtered and that filtrate plated in top agar with *Arthrobacter globiformis* . All phages were purified through three rounds of additional plating. Plaque characteristics are presented in Table 1. Negative stain transmission electron microscopy revealed all three phages to have a siphovirus morphology (Figure 1).

DNA was isolated from lysates prepared for each phage using the Wizard DNA Cleanup Kit (Promega) and prepared for sequencing using the NEB Ultra II FS library prep kit. DNA for phage Fazel was sequenced on an Illumina MiSeq (v.3 reagents), with raw reads subsequently assembled with Newbler (v2.9), and verified using Consed (v29) (Russell, 2018). DNA for phages RustyBoy and Natasha were sequenced on an Illumina NextSeq1000 (XLEAP-P1 kit), with raw reads first trimmed with Cutadapt 4.7 (using the option: –nextseq-trim 30) and filtered with Skewer 0.2.2 (using the options: -q 20 -Q 30 -n -l 50) prior to assembly. The number of reads, depth of sequencing, and genome characteristics are presented in Table 1.

Table 1. Phage Characteristics and Genome Information.

**Table:** 

	**Fazel**	**RustyBoy**	**Natasha**
**GPS**	34.06532 N 106.906919 W	34.066472 N 106.903833 W	34.066472 N 106.903833 W
**Tail Length, nm**	242 - 295 (n=4)	232 - 293 (n=4)	216 - 253 (n=3)
**Capsid Diameter, nm**	60 - 75 (n=4)	62 - 70 (n=4)	63 - 68 (n=3)
**Plaque Size (n=10)**	1.6 – 2.4 mm	0.9 – 1.2 mm	0.7 – 1.1 mm
**Plaque Morphology**	Clear	Cloudy	Clear
**Number of Reads**	110,495 spots	2,563,562 spots	2,654,698 spots
**Sequencing Coverage**	150-base single-end reads	100-base single-end reads	100-base single-end reads
**Genome Length (bp)**	60,088	54,635	54,436
**GC Content**	52.0%	51.4%	51.4%
**Cluster Assignment**	DJ	AW	AW
**Number of Genes**	92	89	88
**Genes with predicted Functions**	29	19	21

Genomes were then annotated using PECAAN v20241104 (Rinehart et al.), which utilized Glimmer v3.02 (Delcher et al., 2007) and GeneMark (v2.5 for Fazel and v2.5p for RustyBoy and Natasha) (Besemer and Borodovsky, 2005) to automatically identify putative genes. Gene start sites were then manually refined using Starterator (http://phages.wustl.edu/starterator/), while putative functions were assigned using HHPred (Zimmerman et al., 2018) searches against the PDB_mmCIF70 (Berman et al., 2000), SCOPe70 (Fox et al., 2014), Pfam-A v37 (Finn et al., 2014), and NCBI_Conserved_Domains databases (Marchler-Bauer et al., 2017), BLASTp searches (Madden, 2013) against the Actinobacteriophage and NCBI non-redundant protein databases, and Phamerator (Cresawn et al., 2011) with the Actino_draft database v578. Default settings were used for all software.

Phages RustyBoy and Natasha are genetically similar, sharing 98 % nucleotide identity. Both phages encode 89/88 predicted genes, 86 of which are conserved between the two phages. Genes *54* and *61* in both phages are sorted into different gene families or “phams” based on limited protein sequence similarity (Cresawn et al., 2011). Indeed, gene *61* from Natasha is an “orpham”, for which no homologues exist within PhagesDB. Putative functions were identified for a subset of genes, including multiple HNH endonucleases, major capsid and protease fusion protein, and Cas4 exonuclease. Based on gene content similarity of at least 35 % to phages in the actinobacteriophage database, PhagesDB, both phages were assigned to cluster AW. As with other AW phages, all genes in RustyBoy and Natasha are encoded unidirectionally, with no identifiable immunity repressor or integrase functions, suggesting these phages are unable to establish lysogeny. Another AW phage, Sporto, that was isolated in Pittsburgh shared 92 % nucleotide identity to RustyBoy. Through genome alignment and phylogeny based on neighbour-joining trees using the MAFFT webserver (Katoh et al., 2019; Kuraku et al., 2013), Natasha, RustyBoy, and Sporto cluster more closely to one another within the AW cluster, warranting consideration for being grouped as an AW subcluster.

Fazel is predicted to encode 92 genes, 6 of which are orphams and 29 of which are assigned a putative function, including deoxycytidylate deaminase, PnuC-like nicotinamide riboside transporter, Thyx-like thymidylate synthase, VRR-NuC domain protein and Cas4 exonuclease. Fazel is assigned to cluster DJ. Consistent with a majority of DJ phages, lysin A and holin functions are encoded next to each other, with lysin B encoded further downstream. All genes are transcribed unidirectionally, which is true for all members of this cluster apart from phage Schwartz33, which contains one predicted gene of unknown function that is transcribed in the opposite direction. Approximately 8 kbp within the central region of the genome contain multiple repeat motifs seen in other DJ cluster phages. Within this region, Fazel contains one predicted orpham, gene *46* (Henson et al., 2022). Based on the absence of predicted immunity repressor or integrase functions, Fazel and other cluster DJ phages are predicted to be lytic phages.

**Nucleotide sequence accession numbers**

Fazel is available at GenBank with Accession No. PP978862 and Sequence Read Archive (SRA) No. SRX24123904. Natasha is available at GenBank with Accession No. PV876962 and Sequence Read Archive (SRA) No. SRX28943173. RustyBoy is available at GenBank with Accession No. PV876932 andSequence Read Archive (SRA) No. SRX28943179.
